# Crystal structure of a photobiologically active furan­ocoumarin from *Artemisia reticulata*

**DOI:** 10.1107/S2056989016003303

**Published:** 2016-03-04

**Authors:** A. K. Bauri, Sabine Foro, Nhu Quynh Nguyen Do

**Affiliations:** a Bioorganic Division, Bhabha Atomic Research Centre, Trombay, Mumbai 400 085, India; b Clemens Schöpf-Institut für Organische Chemie und Biochemie, Technische Universität Darmstadt, Petersenstrasse 22, D-64287 Darmstadt, Germany; c Accident & Emergency Department, Franco, Vietnamese Hospital, 7-Nguyen, Luong Bang Street, HoChiMinh City, Vietnam

**Keywords:** crystal structure, furan­ocoumarin, oroselone, *Artemisia reticulata*, photobiological property, hydrogen bonding

## Abstract

The title furan­ocoumarin, C_14_H_12_O_4_ [systematic name: 9-hy­droxy-2-(prop-1-en-2-yl)-2,3-di­hydro-7*H*-furo[3,2-*g*]chromen-7-one], crystallizes with two independent mol­ecules (*A* and *B*) in the asymmetric unit. The two mol­ecules differ essentially in the orientation of the propenyl group with respect to the mean plane of the furan­ocoumarin moiety; the O—C(H)—C=C torsion angle is 122.2 (7)° in mol­ecule *A* and −10.8 (11)° in mol­ecule *B*. In the crystal, the *A* and *B* mol­ecules are linked *via* O—H⋯O hydrogen bonds, forming zigzag –*A*–*B*–*A*–*B*– chains propagating along [001]. The chains are reinforced by bifurcated C—H⋯(O,O) hydrogen bonds, forming ribbons which are linked *via* C—H⋯π and π–π inter­actions [inter­centroid distance = 3.602 (2) Å], forming a three-dimensional structure.

## Abstract 2

The title furan­ocoumarin, isolated from the Indian herb *A. reticulata*, crystallizes with two independent mol­ecules (*A* and B) in the asymmetric unit. The two mol­ecules differ essentially in the orientation of the propenyl group at the 2-position with respect to the mean plane of the furan­ocoumarin moiety. In the crystal, the two mol­ecules are linked *via* O—H⋯O hydrogen bonds forming zigzag –*A*–*B*–*A*–*B*– chains propagating along [001].

## Chemical context   

The title furan­ocoumarin was isolated from the Indian herb *A. reticulata*, by column chromatography over silica gel with a mixture of binary solvent hexane and ethyl acetate by gradient elution. Furan­ocoumarins, such as oroselone [systematic name: 8-(prop-1-en-2-yl)-2*H*-furo[2,3-*h*]chromen-2-one], whose atomic connectivity has been established by spectrometric and spectroscopic analyses (Schroeder *et al.*, 1959 ▸; Dorofeenko *et al.*, 1973 ▸) but not yet by single crystal X-ray diffraction, exhibit photobiological activity. For example such compounds are employed as photoprotective agents to prevent absorption of harmful UV radiation (Chen *et al.*, 2007 ▸, 2009 ▸). Anti-oxidant and anti-inflammatory activities have also been reported for furano as well as pyrano coumarins and their derivatives (Appendino *et al.*, 2004 ▸; Scott *et al.*, 1976 ▸).
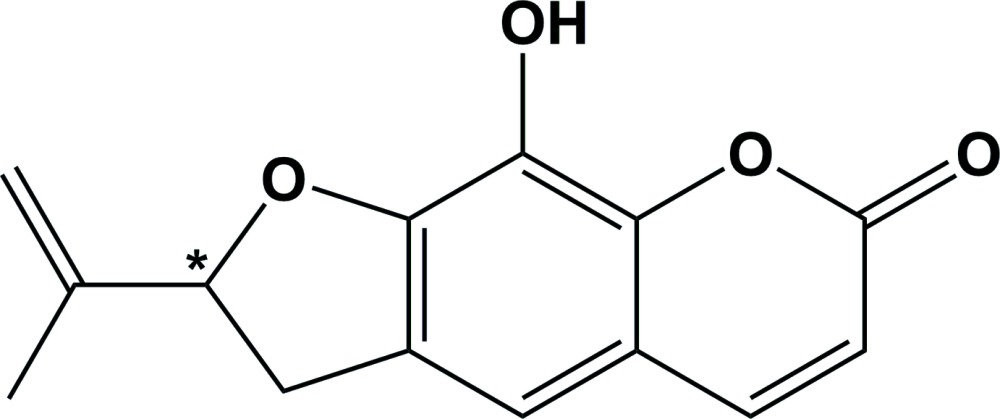


## Structural commentary   

**Figure 1:**
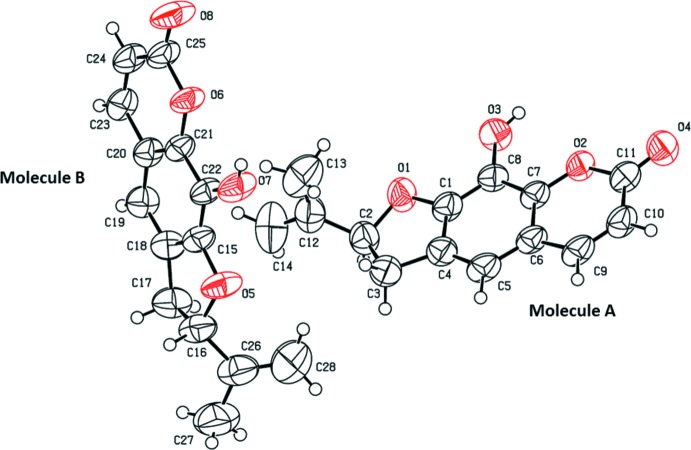
The mol­ecular structure of the two independent mol­ecules (*A* and *B*) of the title compound, showing the atom labelling. Displacement ellipsoids are drawn at the 50% probability level.

**Figure 2:**
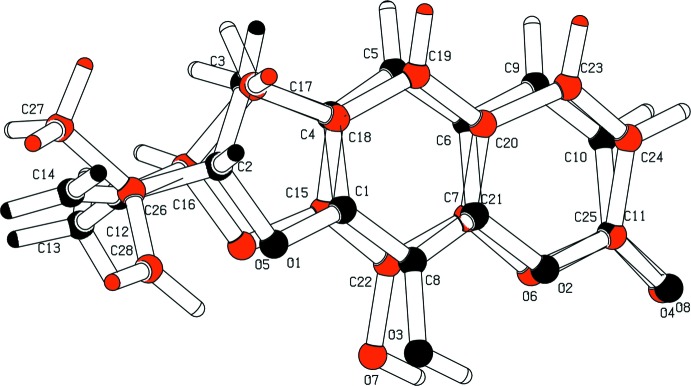
The mol­ecular fit (Spek, 2009 ▸) of mol­ecules *A* (black) and *B* (red) of the title compound.

The title compound, Fig. 1 ▸, crystallizes with two independent mol­ecules (*A* and *B*) in the asymmetric unit. The compound is composed of three fused rings (furan, benzene and pyrone) with hydroxyl and propenyl substituents at positions 9 and 2, respectively. The furan­ocoumarin moieties are essentially planar with r.m.s. deviations of 0.05 Å for mol­ecule *A* (O1/O2/C1–C11) and 0.079 Å for mol­ecule *B* (O5/O6/C16–C25). The furan ring in mol­ecule *A* has an envelope conformation with atom C2 as the flap, deviating by 0.120 (4) Å from the mean plane of the furan­ocoumarin moiety. In mol­ecule *B*, the furan ring has a twisted conformation on bond C17–C16 with atoms C16 and C17 deviating by −0.232 (6) and 0.076 (6) Å, respectively, from the other atoms of the twisted five-membered ring. The two mol­ecules differ essentially in the orientation of the propenyl group with respect to the mean plane of the furan­ocoumarin moiety, as shown by *AutoMolFit* analysis (Spek, 2009 ▸); see Fig. 2 ▸. The O1—C2—C12=C14 torsion angle is 122.2 (7)° in mol­ecule *A*, while the O5—C16—C26=C28 torsion angle is −10.8 (11) ° in mol­ecule *B*. The bond distances and bond angles in the propenyl side chains (C2,C12–C14 in mol­ecule *A* and C16,C26–C28 in mol­ecule *B*) also differ in the two mol­ecules (Table 1 ▸), probably due to libration and bond rotation. Overall the bond distances and bond angles in the furan­ocoumarin moieties are in good agreement with the corresponding values reported for related structures (Stemple & Watson, 1972 ▸; Gupta *et al.*, 1993 ▸; Singh *et al.* 1995 ▸; Magotra *et al.*, 1995 ▸; Thailambal *et al.*, 1986 ▸; Thailambal & Pattabhi, 1987 ▸, 1985 ▸).

The absolute structure of the mol­ecule in the crystal could not be determined by resonant scattering. In order to determine the chirality at atom C2 (in mol­ecule *A*; C16 in mol­ecule *B*), the circular dichroism (CD) spectrum was measured in a solution of chloro­form at concentration of 1 mg/ml using a cell with path length 1 cm. This CD measurement revealed that the absolute configuration of atom C2 (in mol­ecule *A*; C16 in mol­ecule *B*) is *S*.

## Supra­molecular features   

**Figure 3:**
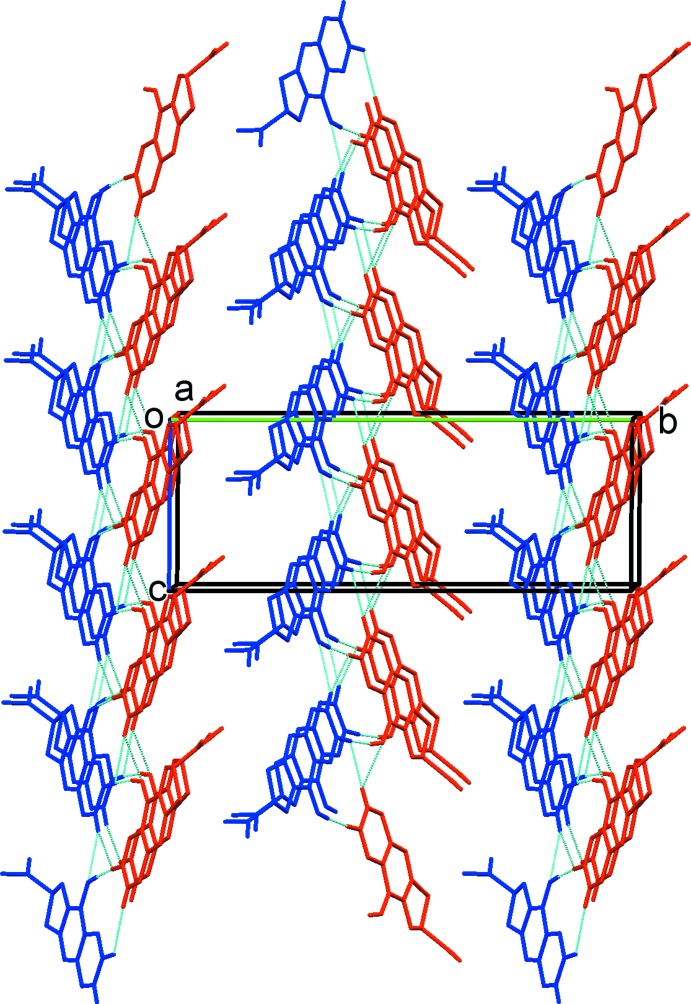
A view along the *a* axis of the crystal packing of the title compound (*A* mol­ecules are blue; *B* mol­ecules are red). The hydrogen bonds are shown as dashed lines (see Table 2 ▸), and C-bound H atoms not involved in hydrogen bonding have been omitted for clarity.

**Figure 4:**
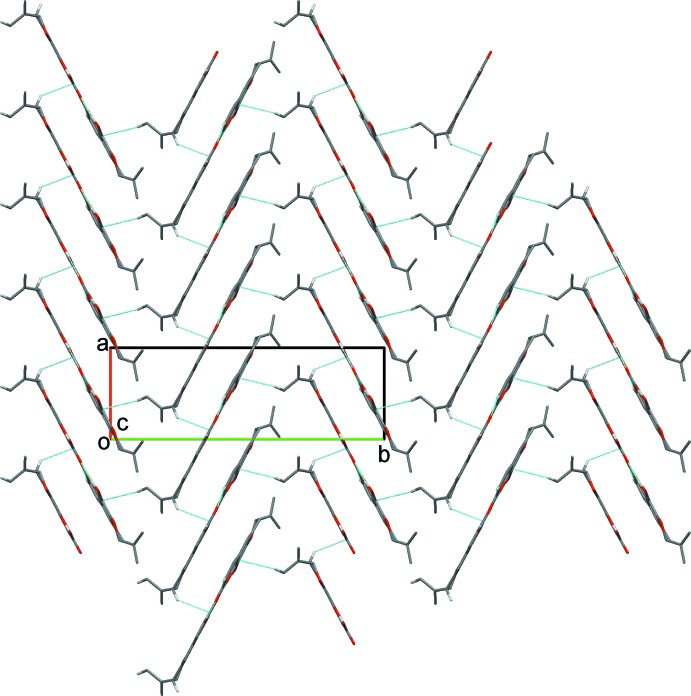
A view along the *c* axis of the crystal packing of the title compound. Hydrogen bonds and C—H⋯π inter­actions are shown as dashed lines (see Table 2 ▸), and C-bound H atoms not involved in hydrogen bonding have been omitted for clarity.

In the crystal, the *A* and *B* mol­ecules are linked *via* O—H⋯O hydrogen bonds, forming zigzag –*A*–*B*–*A*–*B*– chains propagating along the *c-*axis direction; see Table 2 ▸ and Fig. 3 ▸. The chains are reinforced by bifurcated C—H⋯(O,O) hydrogen bonds, forming ribbons (Table 2 ▸ and Fig. 3 ▸). The ribbons are arranged in a herringbone fashion, and are linked *via* C—H⋯π and slipped parallel π–π inter­actions, forming a three-dimensional network; see Fig. 4 ▸ and Table 2 ▸ [*Cg*2⋯*Cg*9^i^ = 3.602 (2) Å, inter­planar distance = 3.4168 (2) Å, slippage 1.284 Å, where *Cg*2 and *Cg*9 are the centroids of rings C1/C4–C8 and C15/C18–C22, respectively; symmetry code: (i) − *x* + 1, *y* + 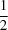, − *z*].

## Database survey   

A search of the Cambridge Structural Database (Version 5.37, update November 2015; Groom & Allen, 2014 ▸) gave 21 hits for the furan­ocoumarin substructure, but only one hit for a 9-hy­droxy furan­ocoumarin, *viz.* 2,3-di­hydro-9-hy­droxy-2-(1-hy­droxy-1-methyl­eth­yl)-7*H*-furo(3,2-*g*)(1) benzo­pyran-7-one monohydrate (refcode FUGVOS; Thailambal & Pattabhi, 1987 ▸).

## Synthesis and crystallization   

The title compound was isolated as a colourless solid from the methanol extract of *A. reticulata* by means of column chromatography over silica gel by gradient elution with a mixture of binary solvents system hexane and ethyl acetate. It was purified by reverse-phase high-pressure liquid chromatography. Colourless rod-like crystals suitable for X ray diffraction analysis were obtained after the title compound was recrystallized three times from ethyl acetate:hexane (1:4) at room temperature by slow evaporation of the solvents (m.p. 498 K). ^1^H NMR data (CHCl_3_, 200 MHz) 7.60 (*d*, 1H, *J* = 9.6 Hz, H-9), 6.85 (*s*, 1H, H-5), 6.20 (*d*, 1H, *J* = 9.6 Hz, H-10), 5.35 (*dd*, 1H, *J* = 8.8 and 8.8 Hz, H-7), 5.11 (*s*, 1H, H_a_-14), 4.94 (*s*, 1H, H_b_-14), 3.47–3.34 (*dd*, 1H, *J* = 9.0 and 1.2 Hz, H_a_-3),3.16–3.04 (*dd*, 1H, *J* = 9.0 and 1.2 Hz, H_b_-3), 1.78 (*s*, 3H, –CH_3_). EIMS (70 ev) data: *m*/*z* (%) 244(15.9) [*M* ^+^], 226 (68.6) [*M* ^+^ − H_2_O), 198 (100) [base peak], 185 (30),171 (16.8), 155 (30.1), 140 (16.4), 127 (13.5), 115 (25.10,85 (11.1), 75 (22.3), 63 (26.5), 41 (16.0).

## Refinement   

Crystal data, data collection and structure refinement details are summarized in Table 3 ▸. The hydroxyl H atoms were located in a difference Fourier map and refined as riding with *U*_iso_(H) = 1.2*U*_eq_(O). The C-bound H atoms were included in calculated positions and treated as riding atoms: C—H = 0.93–0.98 Å with *U*_iso_(H) = 1.2*U*_eq_(C). The limited number of Friedel pairs measured were merged for refinement.

## Supplementary Material

Crystal structure: contains datablock(s) I, global. DOI: 10.1107/S2056989016003303/su5279sup1.cif

Structure factors: contains datablock(s) I. DOI: 10.1107/S2056989016003303/su5279Isup2.hkl

Supporting information file. DOI: 10.1107/S2056989016003303/su5279Isup3.cml

CCDC reference: 1422810

Additional supporting information:  crystallographic information; 3D view; checkCIF report

## Figures and Tables

**Table 1:** Selected geometric parameters (Å, °)

C2—C12	1.500 (8)	C16—C26	1.489 (8)
C12—C14	1.313 (10)	C26—C28	1.363 (13)
C12—C13	1.461 (10)	C26—C27	1.422 (10)
			
C14—C12—C13	122.7 (7)	C28—C26—C27	123.5 (7)
C14—C12—C2	118.9 (7)	C28—C26—C16	121.9 (6)
C13—C12—C2	118.4 (5)	C27—C26—C16	114.7 (6)

**Table 2:** Hydrogen-bond geometry (Å, °) *Cg*2 and *Cg*9 are the centroids of rings O2/C6/C7/C9–C11 and C15–C22, respectively.

*D*—H⋯*A*	*D*—H	H⋯*A*	*D*⋯*A*	*D*—H⋯*A*
O3—H3*O*⋯O8^i^	0.83	1.85	2.676 (5)	174
O7—H7*O*⋯O4^ii^	0.84	1.85	2.671 (5)	168
C10—H10⋯O3^iii^	0.93	2.53	3.199 (5)	129
C10—H10⋯O8^iv^	0.93	2.50	3.415 (6)	166
C24—H24⋯O7^v^	0.93	2.58	3.229 (5)	128
C24—H24⋯O4^vi^	0.93	2.53	3.434 (5)	164
C3—H3*B*⋯*Cg*2^vii^	0.97	2.95	3.871 (5)	160
C13—H13*B*⋯*Cg*9	0.96	2.92	3.680 (9)	137

Symmetry codes: (i) 
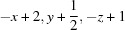
; (ii) 
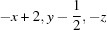
; (iii) 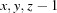; (iv) 
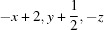
; (v) 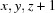; (vi) 
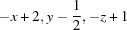
; (vii) 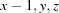.

**Table 3:** Experimental details

Crystal data	
Chemical formula	C_14_H_12_O_4_
*M*_r_	244.24
Crystal system, space group	Monoclinic, *P*2_1_
Temperature (K)	299
*a*, *b*, *c* (Å)	7.2738 (9), 21.426 (2), 8.0152 (9)
β (°)	100.88 (1)
*V* (Å^3^)	1226.7 (2)
*Z*	4
Radiation type	Cu *K*α
μ (mm^−1^)	0.81
Crystal size (mm)	0.50 × 0.18 × 0.15

Data collection	
Diffractometer	Enraf–Nonius CAD-4
Absorption correction	ψ scan (North *et al.*, 1968 ▸)
*T*_min_, *T*_max_	0.688, 0.888
No. of measured, independent and observed [*I* > 2σ(*I*)] reflections	2692, 2133, 1808
*R*_int_	0.111
(sin θ/λ)_max_ (Å^−1^)	0.597

Refinement	
*R*[*F* ^2^ > 2σ(*F* ^2^)], *wR*(*F* ^2^), *S*	0.057, 0.148, 1.08
No. of reflections	2133
No. of parameters	328
No. of restraints	1
H-atom treatment	H-atom parameters constrained
Δρ_max_, Δρ_min_ (e Å^−3^)	0.28, −0.34

Computer programs: *CAD-4-PC* (Enraf–Nonius, 1996 ▸), *REDU4* (Stoe & Cie, 1987 ▸), *SHELXS97* and *SHELXL97* (Sheldrick, 2008 ▸), *PLATON* (Spek, 2009 ▸) and *Mercury* (Macrae *et al.*, 2008 ▸).

## supplementary crystallographic information

### Crystal data

**Table:**

C_14_H_12_O_4_	*F*(000) = 512
*M**_r_* = 244.24	*D*_x_ = 1.322 Mg m^−^^3^
Monoclinic, *P*2_1_	Melting point: 498 K
Hall symbol: P 2y1	Cu *K*α radiation, λ = 1.54180 Å
*a* = 7.2738 (9) Å	Cell parameters from 25 reflections
*b* = 21.426 (2) Å	θ = 6.0–19.8°
*c* = 8.0152 (9) Å	µ = 0.81 mm^−^^1^
β = 100.88 (1)°	*T* = 299 K
*V* = 1226.7 (2) Å^3^	Rod, colourless
*Z* = 4	0.50 × 0.18 × 0.15 mm

### Data collection

**Table:**

Enraf–Nonius CAD-4 diffractometer	1808 reflections with *I* > 2σ(*I*)
Radiation source: fine-focus sealed tube	*R*_int_ = 0.111
Graphite monochromator	θ_max_ = 66.9°, θ_min_ = 4.1°
ω/2θ scans	*h* = −8→8
Absorption correction: ψ scan (North *et al.*, 1968)	*k* = −25→0
*T*_min_ = 0.688, *T*_max_ = 0.888	*l* = −9→2
2692 measured reflections	3 standard reflections every 120 min
2133 independent reflections	intensity decay: 1.0%

### Refinement

**Table:**

Refinement on *F*^2^	Secondary atom site location: difference Fourier map
Least-squares matrix: full	Hydrogen site location: inferred from neighbouring sites
*R*[*F*^2^ > 2σ(*F*^2^)] = 0.057	H-atom parameters constrained
*wR*(*F*^2^) = 0.148	*w* = 1/[σ^2^(*F*_o_^2^) + (0.0983*P*)^2^ + 0.0698*P*] where *P* = (*F*_o_^2^ + 2*F*_c_^2^)/3
*S* = 1.08	(Δ/σ)_max_ = 0.045
2133 reflections	Δρ_max_ = 0.28 e Å^−^^3^
328 parameters	Δρ_min_ = −0.34 e Å^−^^3^
1 restraint	Extinction correction: *SHELXL97* (Sheldrick, 2008), Fc^*^=kFc[1+0.001xFc^2^λ^3^/sin(2θ)]^-1/4^
Primary atom site location: structure-invariant direct methods	Extinction coefficient: 0.0058 (13)

### Special details

**Table:**

Geometry. All e.s.d.'s (except the e.s.d. in the dihedral angle between two l.s. planes) are estimated using the full covariance matrix. The cell e.s.d.'s are taken into account individually in the estimation of e.s.d.'s in distances, angles and torsion angles; correlations between e.s.d.'s in cell parameters are only used when they are defined by crystal symmetry. An approximate (isotropic) treatment of cell e.s.d.'s is used for estimating e.s.d.'s involving l.s. planes.
Refinement. Refinement of *F*^2^ against ALL reflections. The weighted *R*-factor *wR* and goodness of fit *S* are based on *F*^2^, conventional *R*-factors *R* are based on *F*, with *F* set to zero for negative *F*^2^. The threshold expression of *F*^2^ > σ(*F*^2^) is used only for calculating *R*-factors(gt) *etc*. and is not relevant to the choice of reflections for refinement. *R*-factors based on *F*^2^ are statistically about twice as large as those based on *F*, and *R*- factors based on ALL data will be even larger.

### Fractional atomic coordinates and isotropic or equivalent isotropic displacement parameters (Å^2^)

**Table:**

	*x*	*y*	*z*	*U*_iso_*/*U*_eq_	
O1	0.5538 (5)	0.26546 (17)	0.2707 (4)	0.0718 (9)	
O2	1.0199 (4)	0.35147 (14)	0.0003 (3)	0.0584 (7)	
O3	0.8972 (4)	0.32756 (18)	0.2942 (3)	0.0716 (9)	
H3O	0.9962	0.3467	0.3283	0.086*	
O4	1.2459 (5)	0.3904 (2)	−0.1133 (4)	0.0760 (9)	
C1	0.6289 (6)	0.2805 (2)	0.1313 (5)	0.0593 (10)	
C2	0.3653 (7)	0.2416 (2)	0.2102 (6)	0.0677 (11)	
H2	0.2748	0.2741	0.2241	0.081*	
C3	0.3504 (7)	0.2283 (2)	0.0208 (6)	0.0717 (12)	
H3A	0.3550	0.1838	−0.0007	0.086*	
H3B	0.2354	0.2452	−0.0447	0.086*	
C4	0.5186 (6)	0.2609 (2)	−0.0213 (5)	0.0615 (10)	
C5	0.5778 (7)	0.2722 (2)	−0.1707 (5)	0.0646 (11)	
H5	0.5045	0.2598	−0.2732	0.078*	
C6	0.7474 (6)	0.30226 (19)	−0.1693 (5)	0.0574 (10)	
C7	0.8535 (6)	0.32065 (19)	−0.0126 (4)	0.0510 (9)	
C8	0.7961 (6)	0.3100 (2)	0.1404 (5)	0.0559 (10)	
C9	0.8303 (7)	0.3148 (2)	−0.3149 (5)	0.0643 (11)	
H9	0.7672	0.3025	−0.4217	0.077*	
C10	0.9934 (7)	0.3435 (2)	−0.3019 (5)	0.0650 (11)	
H10	1.0412	0.3510	−0.3997	0.078*	
C11	1.0988 (7)	0.3634 (2)	−0.1407 (5)	0.0608 (10)	
C12	0.3366 (8)	0.1867 (3)	0.3189 (7)	0.0784 (14)	
C13	0.4654 (13)	0.1340 (4)	0.3277 (12)	0.120 (2)	
H13A	0.4677	0.1193	0.2150	0.144*	
H13B	0.4243	0.1010	0.3929	0.144*	
H13C	0.5888	0.1471	0.3810	0.144*	
C14	0.1973 (13)	0.1879 (5)	0.4018 (13)	0.131 (3)	
H14A	0.1775	0.1543	0.4697	0.157*	
H14B	0.1184	0.2224	0.3928	0.157*	
O5	0.0856 (5)	0.01320 (19)	−0.0052 (4)	0.0800 (10)	
O6	0.5536 (4)	−0.07278 (16)	0.4256 (3)	0.0632 (8)	
O7	0.4356 (5)	−0.04541 (19)	0.0908 (3)	0.0761 (10)	
H7O	0.5264	−0.0689	0.0873	0.091*	
O8	0.7736 (5)	−0.1141 (3)	0.6163 (4)	0.0985 (14)	
C15	0.1590 (6)	−0.0022 (2)	0.1597 (5)	0.0605 (10)	
C16	−0.1072 (8)	0.0320 (3)	−0.0122 (7)	0.0768 (13)	
H16	−0.1887	−0.0035	−0.0522	0.092*	
C17	−0.1231 (8)	0.0464 (3)	0.1738 (7)	0.0804 (14)	
H17A	−0.1172	0.0909	0.1960	0.097*	
H17B	−0.2382	0.0298	0.2005	0.097*	
C18	0.0452 (6)	0.0134 (2)	0.2722 (6)	0.0648 (11)	
C19	0.1022 (6)	0.0007 (2)	0.4429 (6)	0.0646 (11)	
H19	0.0271	0.0117	0.5200	0.077*	
C20	0.2723 (6)	−0.0287 (2)	0.4982 (5)	0.0555 (9)	
C21	0.3829 (6)	−0.04370 (19)	0.3800 (5)	0.0536 (9)	
C22	0.3273 (6)	−0.0310 (2)	0.2071 (5)	0.0564 (10)	
C23	0.3455 (6)	−0.0475 (2)	0.6713 (5)	0.0617 (10)	
H23	0.2751	−0.0397	0.7547	0.074*	
C24	0.5094 (7)	−0.0754 (2)	0.7141 (5)	0.0670 (12)	
H24	0.5520	−0.0865	0.8270	0.080*	
C25	0.6234 (7)	−0.0890 (2)	0.5911 (5)	0.0658 (11)	
C26	−0.1586 (9)	0.0850 (3)	−0.1320 (8)	0.0882 (16)	
C27	−0.3538 (11)	0.0976 (4)	−0.1745 (13)	0.132 (3)	
H27A	−0.4064	0.0762	−0.2778	0.159*	
H27B	−0.3732	0.1417	−0.1900	0.159*	
H27C	−0.4136	0.0834	−0.0845	0.159*	
C28	−0.0275 (15)	0.1170 (6)	−0.1987 (19)	0.181 (5)	
H28A	−0.0641	0.1490	−0.2764	0.217*	
H28B	0.0986	0.1069	−0.1666	0.217*	

### Atomic displacement parameters (Å^2^)

**Table:**

	*U*^11^	*U*^22^	*U*^33^	*U*^12^	*U*^13^	*U*^23^
O1	0.0756 (18)	0.094 (2)	0.0482 (16)	−0.0229 (17)	0.0165 (13)	−0.0004 (15)
O2	0.0673 (16)	0.0776 (19)	0.0310 (12)	−0.0106 (14)	0.0109 (11)	−0.0007 (12)
O3	0.0767 (18)	0.107 (2)	0.0293 (13)	−0.0232 (17)	0.0062 (12)	−0.0107 (14)
O4	0.079 (2)	0.104 (2)	0.0474 (16)	−0.0193 (19)	0.0178 (14)	−0.0041 (16)
C1	0.074 (2)	0.066 (2)	0.0362 (19)	−0.007 (2)	0.0076 (17)	−0.0017 (18)
C2	0.072 (3)	0.073 (3)	0.059 (3)	−0.017 (2)	0.014 (2)	−0.003 (2)
C3	0.077 (3)	0.074 (3)	0.059 (2)	−0.016 (2)	0.000 (2)	0.001 (2)
C4	0.070 (2)	0.058 (2)	0.052 (2)	−0.0097 (19)	−0.0007 (18)	−0.0027 (17)
C5	0.087 (3)	0.066 (3)	0.0349 (19)	−0.006 (2)	−0.0039 (18)	−0.0053 (18)
C6	0.075 (3)	0.059 (2)	0.0347 (19)	−0.0034 (19)	0.0009 (16)	−0.0010 (16)
C7	0.067 (2)	0.057 (2)	0.0281 (17)	−0.0028 (18)	0.0071 (14)	−0.0035 (14)
C8	0.065 (2)	0.067 (2)	0.0328 (17)	−0.0066 (19)	0.0005 (16)	−0.0047 (17)
C9	0.092 (3)	0.069 (3)	0.0301 (17)	−0.002 (2)	0.0067 (17)	0.0000 (18)
C10	0.087 (3)	0.074 (3)	0.0349 (19)	−0.007 (2)	0.0131 (19)	0.0024 (18)
C11	0.075 (3)	0.073 (3)	0.0370 (19)	0.001 (2)	0.0156 (18)	−0.0007 (17)
C12	0.089 (3)	0.081 (3)	0.067 (3)	−0.022 (3)	0.021 (3)	−0.003 (2)
C13	0.146 (6)	0.094 (5)	0.120 (6)	0.009 (5)	0.023 (5)	0.028 (4)
C14	0.140 (6)	0.125 (6)	0.144 (8)	−0.029 (5)	0.066 (6)	0.018 (5)
O5	0.087 (2)	0.104 (3)	0.0412 (15)	0.024 (2)	−0.0062 (14)	0.0061 (15)
O6	0.0667 (16)	0.092 (2)	0.0293 (13)	0.0173 (15)	0.0059 (11)	0.0098 (13)
O7	0.085 (2)	0.111 (3)	0.0339 (14)	0.0274 (19)	0.0166 (13)	0.0096 (15)
O8	0.091 (2)	0.156 (4)	0.0462 (18)	0.048 (3)	0.0075 (16)	0.026 (2)
C15	0.069 (2)	0.069 (2)	0.039 (2)	0.004 (2)	−0.0011 (17)	0.0035 (18)
C16	0.080 (3)	0.079 (3)	0.063 (3)	0.008 (3)	−0.009 (2)	0.001 (2)
C17	0.073 (3)	0.092 (3)	0.074 (3)	0.019 (3)	0.006 (2)	−0.003 (3)
C18	0.065 (2)	0.069 (3)	0.057 (3)	0.006 (2)	0.0030 (19)	−0.003 (2)
C19	0.069 (2)	0.074 (3)	0.052 (2)	0.005 (2)	0.0156 (19)	−0.006 (2)
C20	0.066 (2)	0.063 (2)	0.0377 (19)	−0.0015 (19)	0.0103 (16)	−0.0041 (16)
C21	0.065 (2)	0.062 (2)	0.0330 (18)	0.0021 (18)	0.0053 (15)	0.0023 (15)
C22	0.067 (2)	0.067 (2)	0.0334 (18)	0.0069 (19)	0.0049 (16)	0.0051 (16)
C23	0.081 (3)	0.075 (3)	0.0317 (18)	0.000 (2)	0.0183 (17)	−0.0042 (17)
C24	0.082 (3)	0.085 (3)	0.0320 (19)	0.002 (2)	0.0072 (18)	0.0045 (19)
C25	0.076 (3)	0.088 (3)	0.0319 (18)	0.012 (2)	0.0070 (18)	0.0117 (18)
C26	0.096 (4)	0.068 (3)	0.087 (3)	0.010 (3)	−0.016 (3)	0.000 (3)
C27	0.111 (5)	0.114 (6)	0.152 (7)	0.017 (4)	−0.025 (5)	0.034 (5)
C28	0.136 (7)	0.150 (8)	0.241 (13)	−0.008 (7)	−0.003 (8)	0.106 (9)

### Geometric parameters (Å, º)

**Table:**

O1—C1	1.371 (5)	O5—C15	1.369 (5)
O1—C2	1.459 (5)	O5—C16	1.449 (6)
O2—C7	1.366 (5)	O6—C25	1.373 (5)
O2—C11	1.384 (5)	O6—C21	1.375 (5)
O3—C8	1.363 (5)	O7—C22	1.365 (5)
O3—H3O	0.8294	O7—H7O	0.8354
O4—C11	1.199 (5)	O8—C25	1.200 (6)
C1—C8	1.361 (6)	C15—C22	1.360 (6)
C1—C4	1.395 (6)	C15—C18	1.376 (7)
C2—C12	1.500 (8)	C16—C26	1.489 (8)
C2—C3	1.529 (7)	C16—C17	1.549 (8)
C2—H2	0.9800	C16—H16	0.9800
C3—C4	1.501 (7)	C17—C18	1.502 (7)
C3—H3A	0.9700	C17—H17A	0.9700
C3—H3B	0.9700	C17—H17B	0.9700
C4—C5	1.369 (7)	C18—C19	1.379 (6)
C5—C6	1.389 (7)	C19—C20	1.384 (6)
C5—H5	0.9300	C19—H19	0.9300
C6—C7	1.400 (5)	C20—C21	1.391 (6)
C6—C9	1.437 (7)	C20—C23	1.447 (5)
C7—C8	1.387 (6)	C21—C22	1.395 (5)
C9—C10	1.322 (7)	C23—C24	1.320 (7)
C9—H9	0.9300	C23—H23	0.9300
C10—C11	1.437 (6)	C24—C25	1.433 (7)
C10—H10	0.9300	C24—H24	0.9300
C12—C14	1.313 (10)	C26—C28	1.363 (13)
C12—C13	1.461 (10)	C26—C27	1.422 (10)
C13—H13A	0.9600	C27—H27A	0.9600
C13—H13B	0.9600	C27—H27B	0.9600
C13—H13C	0.9600	C27—H27C	0.9600
C14—H14A	0.9300	C28—H28A	0.9300
C14—H14B	0.9300	C28—H28B	0.9300
			
C1—O1—C2	107.8 (3)	C15—O5—C16	107.6 (4)
C7—O2—C11	121.7 (3)	C25—O6—C21	121.5 (3)
C8—O3—H3O	136.1	C22—O7—H7O	136.1
C8—C1—O1	123.7 (3)	C22—C15—O5	123.1 (4)
C8—C1—C4	123.2 (4)	C22—C15—C18	123.2 (4)
O1—C1—C4	113.1 (4)	O5—C15—C18	113.7 (4)
O1—C2—C12	107.9 (4)	O5—C16—C26	111.1 (5)
O1—C2—C3	106.3 (4)	O5—C16—C17	105.4 (3)
C12—C2—C3	116.1 (4)	C26—C16—C17	114.4 (5)
O1—C2—H2	108.8	O5—C16—H16	108.6
C12—C2—H2	108.8	C26—C16—H16	108.6
C3—C2—H2	108.8	C17—C16—H16	108.6
C4—C3—C2	103.2 (3)	C18—C17—C16	102.1 (4)
C4—C3—H3A	111.1	C18—C17—H17A	111.3
C2—C3—H3A	111.1	C16—C17—H17A	111.3
C4—C3—H3B	111.1	C18—C17—H17B	111.3
C2—C3—H3B	111.1	C16—C17—H17B	111.3
H3A—C3—H3B	109.1	H17A—C17—H17B	109.2
C5—C4—C1	119.3 (4)	C15—C18—C19	119.8 (4)
C5—C4—C3	133.1 (4)	C15—C18—C17	107.5 (4)
C1—C4—C3	107.6 (4)	C19—C18—C17	132.6 (4)
C4—C5—C6	120.0 (4)	C18—C19—C20	119.4 (4)
C4—C5—H5	120.0	C18—C19—H19	120.3
C6—C5—H5	120.0	C20—C19—H19	120.3
C5—C6—C7	118.4 (4)	C19—C20—C21	118.9 (4)
C5—C6—C9	126.0 (4)	C19—C20—C23	125.3 (4)
C7—C6—C9	115.5 (4)	C21—C20—C23	115.7 (4)
O2—C7—C8	115.0 (3)	O6—C21—C20	122.2 (3)
O2—C7—C6	122.3 (3)	O6—C21—C22	115.5 (3)
C8—C7—C6	122.7 (4)	C20—C21—C22	122.3 (4)
O3—C8—C1	120.0 (4)	C15—C22—O7	121.1 (3)
O3—C8—C7	123.6 (4)	C15—C22—C21	116.3 (4)
C1—C8—C7	116.4 (3)	O7—C22—C21	122.6 (4)
C10—C9—C6	122.1 (4)	C24—C23—C20	121.7 (4)
C10—C9—H9	119.0	C24—C23—H23	119.1
C6—C9—H9	119.0	C20—C23—H23	119.1
C9—C10—C11	121.8 (4)	C23—C24—C25	121.7 (4)
C9—C10—H10	119.1	C23—C24—H24	119.2
C11—C10—H10	119.1	C25—C24—H24	119.2
O4—C11—O2	115.6 (4)	O8—C25—O6	115.8 (4)
O4—C11—C10	127.9 (4)	O8—C25—C24	127.0 (4)
O2—C11—C10	116.5 (4)	O6—C25—C24	117.2 (4)
C14—C12—C13	122.7 (7)	C28—C26—C27	123.5 (7)
C14—C12—C2	118.9 (7)	C28—C26—C16	121.9 (6)
C13—C12—C2	118.4 (5)	C27—C26—C16	114.7 (6)
C12—C13—H13A	109.5	C26—C27—H27A	109.5
C12—C13—H13B	109.5	C26—C27—H27B	109.5
H13A—C13—H13B	109.5	H27A—C27—H27B	109.5
C12—C13—H13C	109.5	C26—C27—H27C	109.5
H13A—C13—H13C	109.5	H27A—C27—H27C	109.5
H13B—C13—H13C	109.5	H27B—C27—H27C	109.5
C12—C14—H14A	120.0	C26—C28—H28A	120.0
C12—C14—H14B	120.0	C26—C28—H28B	120.0
H14A—C14—H14B	120.0	H28A—C28—H28B	120.0
			
C2—O1—C1—C8	173.1 (5)	C16—O5—C15—C22	169.4 (5)
C2—O1—C1—C4	−8.5 (6)	C16—O5—C15—C18	−9.7 (6)
C1—O1—C2—C12	139.0 (4)	C15—O5—C16—C26	142.2 (4)
C1—O1—C2—C3	13.8 (5)	C15—O5—C16—C17	17.7 (6)
O1—C2—C3—C4	−13.6 (5)	O5—C16—C17—C18	−18.6 (6)
C12—C2—C3—C4	−133.6 (5)	C26—C16—C17—C18	−141.0 (5)
C8—C1—C4—C5	−0.9 (7)	C22—C15—C18—C19	1.1 (7)
O1—C1—C4—C5	−179.3 (4)	O5—C15—C18—C19	−179.8 (4)
C8—C1—C4—C3	177.8 (5)	C22—C15—C18—C17	177.9 (5)
O1—C1—C4—C3	−0.6 (6)	O5—C15—C18—C17	−3.1 (6)
C2—C3—C4—C5	−172.7 (5)	C16—C17—C18—C15	13.4 (6)
C2—C3—C4—C1	8.8 (5)	C16—C17—C18—C19	−170.5 (5)
C1—C4—C5—C6	0.9 (7)	C15—C18—C19—C20	−1.1 (7)
C3—C4—C5—C6	−177.5 (5)	C17—C18—C19—C20	−176.9 (5)
C4—C5—C6—C7	−0.4 (6)	C18—C19—C20—C21	1.0 (7)
C4—C5—C6—C9	177.3 (4)	C18—C19—C20—C23	−176.9 (4)
C11—O2—C7—C8	177.6 (4)	C25—O6—C21—C20	0.5 (6)
C11—O2—C7—C6	−3.6 (6)	C25—O6—C21—C22	−178.7 (4)
C5—C6—C7—O2	−178.7 (4)	C19—C20—C21—O6	179.9 (4)
C9—C6—C7—O2	3.4 (6)	C23—C20—C21—O6	−2.0 (6)
C5—C6—C7—C8	0.0 (6)	C19—C20—C21—C22	−1.0 (7)
C9—C6—C7—C8	−177.9 (4)	C23—C20—C21—C22	177.2 (4)
O1—C1—C8—O3	−1.3 (7)	O5—C15—C22—O7	1.2 (7)
C4—C1—C8—O3	−179.6 (4)	C18—C15—C22—O7	−179.8 (4)
O1—C1—C8—C7	178.7 (4)	O5—C15—C22—C21	−180.0 (4)
C4—C1—C8—C7	0.5 (7)	C18—C15—C22—C21	−1.0 (7)
O2—C7—C8—O3	−1.2 (6)	O6—C21—C22—C15	−179.9 (4)
C6—C7—C8—O3	−180.0 (4)	C20—C21—C22—C15	0.9 (6)
O2—C7—C8—C1	178.8 (4)	O6—C21—C22—O7	−1.1 (6)
C6—C7—C8—C1	−0.1 (6)	C20—C21—C22—O7	179.7 (4)
C5—C6—C9—C10	−179.6 (5)	C19—C20—C23—C24	180.0 (5)
C7—C6—C9—C10	−1.9 (7)	C21—C20—C23—C24	2.0 (6)
C6—C9—C10—C11	0.5 (7)	C20—C23—C24—C25	−0.5 (8)
C7—O2—C11—O4	−179.5 (4)	C21—O6—C25—O8	−180.0 (5)
C7—O2—C11—C10	2.0 (6)	C21—O6—C25—C24	1.1 (7)
C9—C10—C11—O4	−178.8 (5)	C23—C24—C25—O8	−179.9 (6)
C9—C10—C11—O2	−0.5 (7)	C23—C24—C25—O6	−1.1 (8)
O1—C2—C12—C14	122.2 (7)	O5—C16—C26—C28	−10.8 (11)
C3—C2—C12—C14	−118.7 (7)	C17—C16—C26—C28	108.4 (10)
O1—C2—C12—C13	−57.9 (7)	O5—C16—C26—C27	167.1 (6)
C3—C2—C12—C13	61.3 (7)	C17—C16—C26—C27	−73.7 (8)

### Hydrogen-bond geometry (Å, º)

Cg2 and Cg9 are the centroids of rings O2/C6/C7/C9–C11 and C15–C22, respectively.

**Table:**

*D*—H···*A*	*D*—H	H···*A*	*D*···*A*	*D*—H···*A*
O3—H3*O*···O8^i^	0.83	1.85	2.676 (5)	174
O7—H7*O*···O4^ii^	0.84	1.85	2.671 (5)	168
C10—H10···O3^iii^	0.93	2.53	3.199 (5)	129
C10—H10···O8^iv^	0.93	2.50	3.415 (6)	166
C24—H24···O7^v^	0.93	2.58	3.229 (5)	128
C24—H24···O4^vi^	0.93	2.53	3.434 (5)	164
C3—H3*B*···*Cg*2^vii^	0.97	2.95	3.871 (5)	160
C13—H13*B*···*Cg*9	0.96	2.92	3.680 (9)	137

Symmetry codes: (i) −*x*+2, *y*+1/2, −*z*+1; (ii) −*x*+2, *y*−1/2, −*z*; (iii) *x*, *y*, *z*−1; (iv) −*x*+2, *y*+1/2, −*z*; (v) *x*, *y*, *z*+1; (vi) −*x*+2, *y*−1/2, −*z*+1; (vii) *x*−1, *y*, *z*.
